# Neural Network Aided Detection of Huntington Disease

**DOI:** 10.3390/jcm11082110

**Published:** 2022-04-10

**Authors:** Gerardo Alfonso Perez, Javier Caballero Villarraso

**Affiliations:** 1 Department of Biochemistry and Molecular Biology, University of Cordoba, 14071 Cordoba, Spain; bc2cavij@uco.es; 2 Biochemical Laboratory, Reina Sofia University Hospital, 14004 Cordoba, Spain

**Keywords:** Huntington disease, DNA methylation, neural networks

## Abstract

Huntington Disease (HD) is a degenerative neurological disease that causes a significant impact on the quality of life of the patient and eventually death. In this paper we present an approach to create a biomarker using as an input DNA CpG methylation data to identify HD patients. DNA CpG methylation is a well-known epigenetic marker for disease state. Technological advances have made it possible to quickly analyze hundreds of thousands of CpGs. This large amount of information might introduce noise as potentially not all DNA CpG methylation levels will be related to the presence of the illness. In this paper, we were able to reduce the number of CpGs considered from hundreds of thousands to 237 using a non-linear approach. It will be shown that using only these 237 CpGs and non-linear techniques such as artificial neural networks makes it possible to accurately differentiate between control and HD patients. An underlying assumption in this paper is that there are no indications suggesting that the process is linear and therefore non-linear techniques, such as artificial neural networks, are a valid tool to analyze this complex disease. The proposed approach is able to accurately distinguish between control and HD patients using DNA CpG methylation data as an input and non-linear forecasting techniques. It should be noted that the dataset analyzed is relatively small. However, the results seem relatively consistent and the analysis can be repeated with larger data-sets as they become available.

## 1. Introduction

Huntington disease (HD) is a neurological progressive disorder [1,2,3,4]. The typical onset of the illness is in mid-adult life [5,6,7] causing uncontrolled movements as well as declining cognitive and reasoning skills. The disease is associated with a mutation of a gene in Chromosome 4 [8,9,10] related to the gene encoding for the protein huntingtin [11,12,13]. There are also other proteins associated with the illness. Vonsattel [14] estimates that death typically occurs approximately 12 to 15 years after the onset of symptoms but some other authors have mentioned a slightly longer period, approximately 15 to 20 years [15,16].

Ross [17] identified three clinical stages of the disease: (1) early-stage, (2) middle-stage and (3) late-state. In the early-stage phase the symptoms are relatively minor with some moderate decrease in motor skills (including some involuntary movements) as well as increased irritability. In the middle-stage phase typically the symptoms are more apparent with a visible decrease in motor and cognitive skills. The late-stage is the third and final stage. In this phase the patient tends to have severe reduction in motor and cognitive skills with in many cases the patient unable to leave the bed or communicate. Regrettably, there is no cure for HD.

Currently there is genetic testing available for HD [18,19,20], which is typically only carried out when there is significant clinical evidence or family history suggesting the presence of HD. There are also economic costs to take into account when carrying out tests. This paper presents a complementary approach for the detection of HD using DNA methylation data [21,22,23]. DNA methylation data has been associated with many diseases, particularly in illnesses such as different types of cancers. DNA methylation analysis is a relatively inexpensive and simple technique.

**Figure 1:**
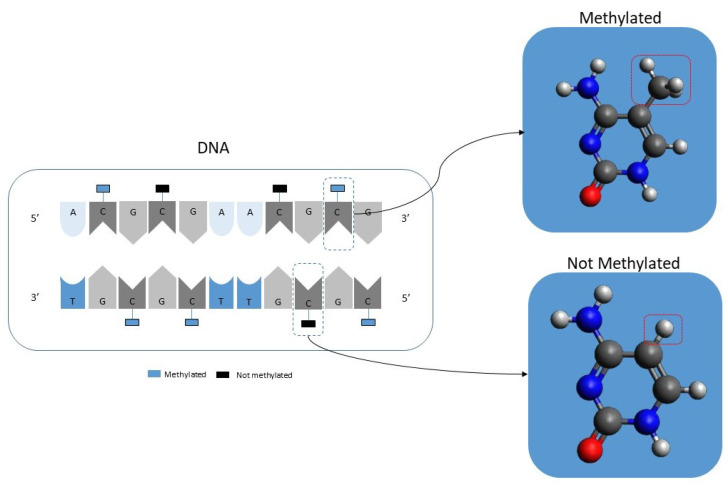
Illustration showing the concept of DNA methylation.

In simple terms, DNA CpG methylation consists of the addition of a methyl group to a cytosine-phosphate-guanine group as illustrated in Figure 1. DNA methylation is a well-known epigenetic change [24,25,26]. Current laboratory equipment can quickly analyze more than 450,000 CpGs per patient. It should be noted that the resulting data will consist of a percentage value ranging from 1, meaning that it is fully methylated, to 0, meaning that it is entirely unmethylated. It should also be noted that there is a new generation of equipment that can analyze in excess of 800,000 but this equipment is not yet as widely used as the 450,000 CpGs equipment.

There is a significant amount of literature using DNA CpG methylation data in fields such as aging [27,28,29], cancer [30,31,32], Alzheimer [33,34,35] and Multiple Sclerosis [36]. A common approach in the existing literature is trying to identify relevant CpGs using linear methods. However, in principle there is no indication that the underlying DNA methylation process of aging or of any these illnesses needs to follow a linear behaviour. There are some papers using non-linear methods. For instance, Vidaki [37] analyzed DNA methylation data using neural networks for forensic age purposes. Marchevsky [38] used a similar approach but in this case applied to the classification of different types of lung cancers. In fact, one of the most frequent applications is in the classification of different types of cancers or in differentiating between control and cancer patients [39,40,41,42,43,44]. This approach has also been applied in the context of some neurological illnesses, such as Alzheimer [45,46].

Huntington disease has attracted less interest in the existing literature than other neurological diseases such as Alzheimer. However, there are some interesting articles exploring the disease in the context of DNA methylation [47,48,49]. To the best of the knowledge of the authors of this article, the existing literature covering Huntington in the context of DNA methylation follows a linear approach.

## 2. Aims

One of the main aims of this article is to provide alternative approaches to detect Huntington Disease using available and relatively straightforward techniques based on DNA methylation. Currently there are no treatments for HD but we are relatively optimistic that eventually there will be some treatment break through. It is acknowledged that there remains significant technical hurdles but when treatments are developed it would be useful to have techniques for screening.

## 3. Materials and Methods

A classification variable $Y_{i}$ was defined for each case as follows.

(1) $$y_{i}=\left\{\begin{array}{c} 0\;\;if\;\;Control\\ 1\;\;if\;\;Huntington \end{array}\right.$$

Therefore for m cases analyzed there is a vector *Y*

(2) $$Y=\{y_{1},\ldots .,y_{m}\}$$

There is also an associated vector for each variable $y_{i}$ containing the methylation levels for n CpGs.

(3) $$X_{p}=\left\{\begin{array}{c} \;X_{p}^{1}\\ \;X_{p}^{2}\\ .\\ .\\ .\\ \;X_{p}^{n} \end{array}\right\}$$

Hence, the dataset can be visualized as follows:

(4) $$\left(\begin{array}{cccc} \;y_{1} & y_{2} & \ldots & y_{m}\\ \;X_{1}^{1} & X_{2}^{1} & \ldots & X_{m}^{1}\\ \;X_{1}^{2} & X_{2}^{2} & \ldots & X_{m}^{2}\\ . & . & \; & .\\ . & . & \; & .\\ . & . & \; & .\\ \;X_{1}^{n} & X_{2}^{n} & \ldots & X_{m}^{n} \end{array}\right)$$

The dimensionality of the problem can be defined as *n*.

### 3.1. Algorithm

First, the dimensionality of the dataset is reduced. Each CpG is used (individually) as an input for a classification algorithm. The steps are as follows:

1. Select a classification algorithm φ using each CpG (individually) as an input and the classification variable as output $\varphi (X^{i},y)$. In this notation $X^{i}$ refers to the vector containing the methylation data for all the cases analyzed for a single CpG.
  (5) $$x^{i}=\left\{X_{1}^{i},X_{2}^{i},\ldots ,X_{m}^{i}\right\}$$
2. Separate the data into a training and a testing dataset. For clarity purposes the training and testing datasets are labeled *A* and *B* respectively.
  (6) $$A=\left(\begin{array}{cccc} \;y_{1} & y_{2} & \ldots & y_{k}\\ \;X_{1}^{1} & X_{2}^{1} & \ldots & X_{k}^{1}\\ \;X_{1}^{2} & X_{2}^{2} & \ldots & X_{k}^{2}\\ . & . & \; & .\\ . & . & \; & .\\ . & . & \; & .\\ \;X_{1}^{n} & X_{2}^{n} & \ldots & X_{k}^{n} \end{array}\right)$$
  (7) $$B=\left(\begin{array}{cccc} \;y_{k+1} & y_{k+2} & \ldots & y_{m}\\ \;X_{k+1}^{1} & X_{k+2}^{1} & \ldots & X_{m}^{1}\\ \;X_{k+1}^{2} & X_{k+2}^{2} & \ldots & X_{m}^{2}\\ . & . & \; & .\\ . & . & \; & .\\ . & . & \; & .\\ \;X_{k+1}^{n} & X_{k+2}^{n} & \ldots & X_{m}^{n} \end{array}\right)$$
3. Train the non-linear algorithm with the training dataset (φ(A)).
4. Estimate classification forecasts
  (8) $$YP=\{YP_{k+1},\ldots ,YP_{m}\}$$
  using the testing dataset and the trained algorithm (φ(B))
5. Estimate the accuracy of the forecast (YP) comparing it with the actual values $\left\{y_{k+1},y_{k+2},\ldots ,y_{m}\right\}$
  (a) For l=k+1 to m
    (9) $$if\;\left\{\begin{array}{c} \;YP_{l}=Y_{l}\;then\;a_{l}=1\\ else\;a_{l}=0 \end{array}\right.$$
  (b) Estimate the accuracy
    (10) $$F^{i}=\left\{\sum_{l=k+1}^{m}a_{l}\right\}\frac{1}{\left(m-k\right)}$$
6. Repeat steps 2 to 5, *k* times.
7. Estimate the average of the accuracy $\left\{F_{1}^{i},\ldots ,F_{k}^{i}\right\}$.
  (11) $$MF^{i}=\frac{1}{k}\sum F^{i}$$
8. Repeat steps 1 to 8 (estimating forecasting accuracy individually for each CpG).
  (12) $$MF=\{MF^{1},\ldots ,MF^{n}\}$$
9. Define a cut off level ($MF_{c}$).
10. Exclude from the analysis all $MF^{i}<MF_{c}$.
11. Create a new list of CpGs according to the condition shown in the previous step.
  (13) $$MF^{new}=\left\{MF_{*}^{1},\ldots ,MF_{*}^{nn}\right\}\;with\;nn\leq n.$$
  Note: the dimensionality has been reduced from *n* to nn.

In the second part of the algorithm a combinatorial approach was followed. The starting point of this second part is the already filtered CpG list with the previously mentioned dimensionality reduction from n to nn. The steps of the second part are as follows:

1. Starting with the reduced list of CpGs. As an example, patient *p* will now have associated the following CpGs.
  (14) $$\left\{\begin{array}{c} \;X_{p}^{*1}\\ \;X_{p}^{*2}\\ .\\ .\\ .\\ \;X_{p}^{*nn} \end{array}\right\}$$
  Notice again the reduction in the dimensionality from *n* to nn (nn < *n*).
2. The data, as in the first part of the algorithm, was divided into a training and a testing datasets denoted this time as $A^{*}$ and $B^{*}$.
  (15) $$A^{*}=\left(\begin{array}{cccc} \;y_{1} & y_{2} & \ldots & y_{k}\\ \;X_{1}^{*1} & X_{2}^{*1} & \ldots & X_{k}^{*1}\\ \;X_{1}^{*2} & X_{2}^{*2} & \ldots & X_{k}^{*2}\\ . & . & \; & .\\ . & . & \; & .\\ . & . & \; & .\\ \;X_{1}^{*nn} & X_{2}^{*nn} & \ldots & X_{k}^{*nn} \end{array}\right)$$
  (16) $$B^{*}=\left(\begin{array}{cccc} \;y_{k+1} & y_{k+2} & \ldots & y_{m}\\ \;X_{k+1}^{*1} & X_{k+2}^{*1} & \ldots & X_{m}^{*1}\\ \;X_{k+1}^{*2} & X_{k+2}^{*2} & \ldots & X_{m}^{*2}\\ . & . & \; & .\\ . & . & \; & .\\ . & . & \; & .\\ \;X_{k+1}^{*nn} & X_{k+2}^{*nn} & \ldots & X_{m}^{*nn} \end{array}\right)$$
3. Train the non-linear algorithm with the training dataset ($\varphi (A^{*})$).
4. Estimate classification forecasts
  (17) $$YP^{*}=\left\{YP_{k+1}^{*},\ldots ,YP_{m}^{*}\right\}$$
  using the reduced testing dataset and the trained algorithm ($\varphi (B^{*})$).
5. Estimate the accuracy of the forecast ($YP^{*}$) comparing it with the actual values $\left\{Y_{k+1},Y_{k+2},\ldots ,Y_{m}\right\}$
  (a) For l=k+1 to m
    (18) $$if\;\left\{\begin{array}{c} \;YP_{l}^{*}=Y_{l}\;then\;a_{l}=1\\ else\;a_{l}=0 \end{array}\right.$$
  (b) Estimate the accuracy
    (19) $$F^{*}=\left\{\sum_{l=k+1}^{m}a_{l}\right\}\frac{1}{\left(m-k\right)}$$
6. Repeat steps 2 to 5, *k* times.
7. Estimate the average of the accuracy $\left\{F_{1}^{*},\ldots ,F_{k}^{*}\right\}$.
  (20) $$MF^{*}=\frac{1}{k}\sum F^{*}$$
8. Reduce the number of of CpGs considered by one (randomly selected). Hence, the dimensionality is reduced from nn to nn-1. As an example, the initial reduced CpG list for patient *p* was:
  (21) $$\left\{\begin{array}{c} \;X_{p}^{*1}\\ \;X_{p}^{*2}\\ .\\ .\\ .\\ \;X_{p}^{*nn} \end{array}\right\}$$
  After this step the new CpG list is:
  (22) $$\left\{\begin{array}{c} \;X_{p}^{**1}\\ \;X_{p}^{**2}\\ .\\ .\\ .\\ \;X_{p}^{**(nn-1)} \end{array}\right\}$$
9. Repeat steps 2 to 5 with the new CpG list (of dimensionality nn−1).
10. Estimate the average ($MF^{**}$) of the accuracy $\left\{F_{1}^{*},\ldots ,F_{k}^{*}\right\}$.
11. Choose between the previous and the current configuration
  (a) If $MF^{**}>MF^{*}$, then accept the CpG list used to obtain $MF^{**}$ as the current best list. $MF^{Current}=MF^{**}$.
  (b) If $MF^{**}\leq MF^{*}$, then reject the CpG list used to obtain $MF^{**}$ and continue using the previous list. $MF^{Current}=MF^{*}$.
12. Repeat steps 8 to 11 until:
  (a) The number of iterations reaches a predetermined level ($iter_{max}$)
    or
  (b) $MF^{current}\leq MF_{p}$, where $MF_{p}$ is a predetermined acceptable value for the accuracy level.

### 3.2. Data

DNA methylation data was obtained from the GEO database with the accession code GSE 147004 [49]. The dataset contains DNA methylation data for 76 samples, including 24 control (healthy), 19 HD pre-manifest and 33 HD manifest. The manifest and the pre-manifest sets were grouped together. The dataset contains 485,512 CpG DNA methylation data per patient. The samples were obtained from blood (buffy coat). Age and body fat index data are also available. As previously mentioned the methylation data is expressed as a percentage value (from 0 to 1) with a value of 1 suggesting full methylation. Healthy (control) cases were assigned the categorical variable 0 while HD patients were assigned the categorical variable 1. For clarity purposes some potential values for “A” are shown below.

(23) $$A=\left(\begin{array}{cccc} 0 & 0 & \ldots & 1\\ 0.651094 & 0.650451 & \ldots & 0.634303\\ 0.960434 & 0.954877 & \ldots & 0.957124\\ . & . & \; & .\\ . & . & \; & .\\ . & . & \; & .\\ 0.077337 & 0.063247 & \ldots & 0.090948 \end{array}\right)$$

where the values in the first row identify healthy cases (with a “0” categorical value) and HD patients (with a “1” categorical value). All the other rows represent the methylation level of different CpGs expressed as a percentage value. For instance, the second row is associated with one CpG (cg00000029), the third row with a different one (cg00001108) and so on. Some DNA methylation values from an illustrative patient can be seen below.

(24) $$\left(\begin{array}{cc} cg00000029 & 0.651094\\ cg00000108 & 0.960434\\ cg00000109 & 0.899284\\ . & .\\ . & .\\ . & . \end{array}\right)$$

### 3.3. Artificial Neural Networks

The classification technique used was an artificial neural network. Neural networks are a flexible approach that have been used successfully in multiple disciplines, including illness identification using DNA methylation data. One of the advantages is that neural networks do not require previous knowledge of the process to be model.It should be noted that the algorithm was constructed in a generic way to allow for the use of other classification techniques. An artificial neural network (ANN) is a well-known technique, inspired by the human brain. The basic component of an ANN is an artificial neuron which in basic terms is a mathematical function translating some input signal into an output signal. The artificial neuron has a related weight associated with it. This weight is a value that it is calibrated during a training phase. There are many training algorithms. The objective of these training algorithms is to minimize the classification error when comparing the actual output value with the output generated by the neural network. Artificial neurons are typically arranged in layers. One critical factor when deciding the architecture of the neural network is to decide the number of layers. In this paper we tested several ANN configurations with the number of layers ranging from 1 to 10. There is no clear definition of the concept of deep learning but it is typically assumed that a neural network with several layers can be considered deep learning. The analysis was carried out, using the standard approach, dividing the dataset in a training dataset and a testing dataset. The training data set contained approximately two thirds of the cases (66.6%) and the testing data set one third (33.3%). Unless otherwise stated the forecasting accuracy refers to than in the testing dataset. Each hidden layer contained 100 sigmoid neurons and the maximum number of iterations was 1000. The analysis was also repeated using only the pre-manifest and control cases (excluding the manifest cases). In this second approach the number of cases is lower. In order to focus on out-of-sample precision the training and the testing data set were divided into two data sets of roughly equal dimensions.

### 3.4. Similarities and Differences with Previously Published Research

Although they differ quite a bit from our field of application, some authors have also carried out a methodological approach similar to that of our study, having used computer-assisted diagnostic strategies for the detection of neurodegenerative diseases. For this purpose, they have used, for example, the pooled analysis of information from clinical information, such as Lones et al. who designed an algorithm based on the collection of information related to movement disorders in patients with Parkinson’s disease (PD). To this end, they performed continuous monitoring of dyskinesia in six patients with PD using a device that comprised a tri-axial accelerometer and tri-axial gryoscope [50]. Other authors have also carried out machine-learning approaches based on diagnostic imaging information, such as Elahifasaee et al., who designed an algorithm for the classification of diagnostic images compatible with Alzheimer’s disease (AD). To do this, they used a feature decomposition and kernel discriminant analysis (KDA) applying it to information from MR brain images from 830 subjects comprising 198 AD patients [51]. Other more recent studies have also carried out a methodological approach more similar to ours, having used strategies based on artificial intelligence for the detection of neurodegenerative diseases, although also based on clinical or neuroimaging information [52,53]. However, very few investigations use this methodology for the design of diagnostic algorithms based on information from molecular studies. Bahado-Singh et al. devised a predictive model for the diagnosis of cerebral palsy using information about DNA epigenetic profiles. These authors are the first to mention the concept of deep learning that we have discussed previously [54]. Something more similar to our research would be the work published a few months ago by Sh et al. because like us, these authors use information from the GEO database. Using a machine-learning model, they have identified the role of natural killer T cells (NKT) and granulocyte macrophage progenitor (GMP) in the aetiology of AD. To do so, they relied on information from mRNA data from blood from 711 subjects, including the control group (238 patients), mild cognitive impairment (189 patients), and AD (284 patients) [55]. Nevertheless, there are no studies with these methodological approaches that are based on epigenetic information and are focused on Huntington’s disease (HD), so the present study would be a first in this regard. In accordance with what we have commented on previously, there are studies that use artificial intelligence formulas as a diagnostic resource, but they are based on information from neuroimaging tests [56,57]. Perhaps the closest thing are studies based of genomic information. Lovrecic et al devised a diagnostic algorithm based on the expression of 12 candidate genes [58]. A decade later, the same research group used machine learning techniques to study these genes and discovered that two of them (ARFGEF2 and GOLGA8G) were significantly up-regulated [59]. All the same, as we initially stated, the use of artificial intelligence strategies based on epigenetic information for the diagnosis of HD was an unprecedented topic until nowadays.

## 4. Results

**Figure 2:**
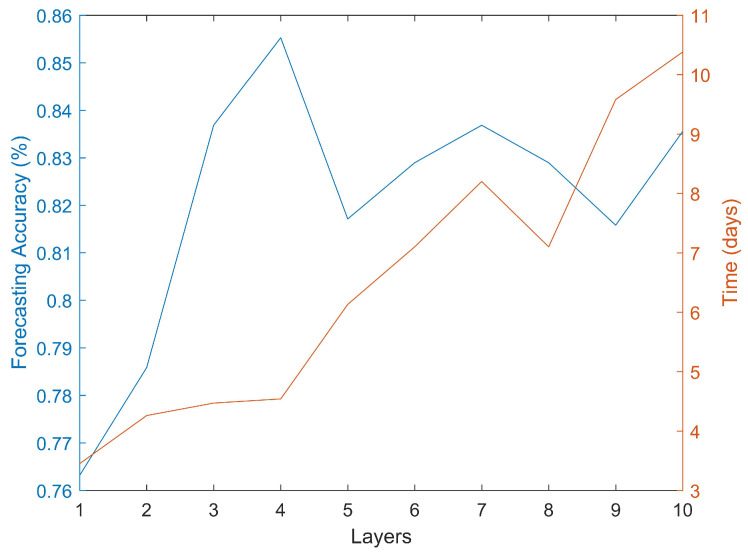
Forecasting accuracy and required computational time using different ANN architectures.

The results for the first part of the algorithm can be seen in Figure 2. The most accurate classifications were obtained when using a four layers ANN. Further increases in the number of layers did not appear to increase the accuracy of the forecasts. It can be seen that the initial increase in the number of layers did improve the accuracy but after reaching four layers the process seems to have reached a plateau. It should be noted that the computational time required to carry out this analysis was rather substantial. For instance, it required 3.45 days to obtain the results for a one-layer ANN architecture and 10.38 days for a 10-layer architecture. The training process, as shown in Table 1, required significant time. However after training, the application to data from a new patient requires negligible time (a few seconds). The scaled conjugate gradient training algorithm generated better forecasts than other training algorithms such as one-step secant backpropagation or resilient backpropagation. All the calculations were done with an Intel(R) Core(TM) i5-4590 3.3 GHz computer. There are some options to reduce the computational type. For instance, the algorithm was designed in order to make it easily parallelizable, particularly the dimensionality reduction part. This algorithm can be distributed in several computers in a cluster with each computer analyzing a different group of CpGs.

The second part of the algorithm further increased the accuracy of the forecasts. The best results are, similar to the previous case, obtained when using an artificial neural network with four layers (Table 1). Deeper artificial neural networks, such as the one using ten hidden layers, did not improve the results obtained using four layers. The sensitivity and specificity (Table 2) were 0.95 and 0.80 respectively with a final list of 237 CpGs, representing a very substantial reduction from the initial 485,512 available CpGs. The complete 237 CpG list can be found in the supplementary material. Controlling for age and body mass index did not impact the classifications obtained.

## 5. Discussion

Huntington disease is a degenerative illness currently without a cure. However, it is an area of very active research and it is possible that in the future there will be some treatments. Currently there are some specific genetic tests that can identify the illness however they are typically only prescribed when there are clear indications of the illness such as clinical evidences or family history. When treatments become available it is likely that early detection becomes crucially important. In this regard it would be interesting to be able to detect the illness in general blood tests as early as possible. Blood DNA methylation data can be obtained through an inexpensive a relatively quick test that can be carried out and used to test for indications of multiple different illness, such as cancer, and it is likely that in the future this type of test will become more widespread. Using the same basic blood DNA methylation data when testing for other illnesses it may be possible to test for indications of HD as well.

Increasing our understanding of the DNA methylation dynamics in the context of Huntington, such as for instance identifying relevant CpGs as well as improving our search algorithms, can encourage other researchers to obtain more DNA methylation data which in turn can be used to develop more accurate models, in this way creating a positive feedback loop. This is particularly important because while there is a significant existing body of research covering the topic there is much less research than in other degenerative neurological diseases, such as Alzheimer.

From a computational point of view the results show that increasing the complexity of the models beyond a certain point did not translate into an increase in the forecasting accuracy. The best results were obtained using four layers. It is however possible that, using larger datasets, the complexity of the models i.e., the number of layers, might need to be further increased but there is clearly an upper limit. There is also a clear trade-off between the complexity of the model and the required computational time, with some of the models tested requiring in excess of ten days of computing power. Controlling for age and body mass composition did not appear to change the forecasts. However, this might be due to a relatively small data set.

The case of pre-manifest cases was also analyzed independently. It was shown that the accuracy of the classification was relatively high when using only pre-manifest and control cases (excluding HD manifest cases). It should be noted that the accuracy when using this approach (pre-manifest and control only) was high, but lower than that obtained using all cases (control, pre-manifest and manifest), which might be due to a relatively small sample size.

## 6. Future Research and Limitations

As a line of future research it will be interesting to have access to large data sets that will likely help further improving the accuracy of the model. The relatively small size of the data pool is one of the limitations of this paper. It would be interesting to have reasonably large sets of data at different stages of the illness (not only pre-manifest and manifest) in order to identify the progressions. This systematic, machine-learning driven approach, may prove to be important when comparing different types of potential future medications and their impact on the progression of the illness with quantifiable changes in the level of DNA methylation.

It might be possible to carry out the same type of analysis using some non-invasive biomarkers such as saliva or urine, rather than blood. This will have certain advantages with less discomfort for patients and easier collection. So far we have not found data linking DNA methylation in saliva or urine to HD but it is possible that it can be successfully used to determine the presence of the illness. Based on the experience with other illnesses it is likely that there is a different DNA methylation pattern. This would be another interesting line of future research.

The presented approach to identify relevant combinations of CpGs can be used for other diseases, as long as there is existing DNA methylation data. Similarly, the algorithm was designed to allow for other training techniques besides artificial neural networks. This is potentially an interesting area of future research.

Another very interesting area of future research is longitudinal analysis. Analyzing DNA methylation changes as the illness progress could be used to quantitatively map the progression of the illness. Another important application of longitudinal analysis, after the above mentioned mapping is created, is as a quantitative measure of the impact of potential treatments in the progression of the illness. This is a very promising field of research but unfortunately there is currently not enough data available to be carried out and would ideally require the monitor of patients over extended periods of time. Longitudinal analysis could potentially greatly help enhancing the knowledge of the progression of the illness. Artificial intelligence techniques, such as neural networks, could be a very interesting tool for analyzing this type of complex and data driven analysis.

## 7. Conclusions

Huntington disease is a devastating illness. There are several research groups working on potential treatments for this illness but as of now there is no cure. We are cautiously confident that eventually there will be a treatment. As previously mentioned, we do not suggest carry out mass screening at the moment, but when a treatment is developed it will likely be important to have ways to detect the illness, particularly when using general test in patients that might be asymptomatic. It is likely that when such treatment arises early detection will be important. In this scenario, of a treatment available, such a tool could be used as pre-screening with the healthcare professional taking care of the patient to decide if it is appropriate to refer the patient to a specialist or to carry out further testing such as DNA sequencing. In this scenario extreme care should be taken when communicating with the patient, explaining clearly that the test has a degree of uncertainty and that the diagnosis is not yet confirmed. This is, once more, in the context of a potential treatment developed for the illness. The objective is to try to detect the illness as soon as possible (to increase the chances of a successful treatment) while at the same time minimizing the potential physiological impact on the patient.

## Figures and Tables

**Table 1:** Forecasting precision obtained with the different neural network configurations (after the second part of the algorithm). The second column shows the results using control, pre-manifest and manifest cases while the third column includes only control and pre-manifest cases. The fourth column shows the computational time required for training the neural network.

N. Layers	Max Precision	Max Precision	Training Time
	(Control & Manifest & Pre-Manifest)	(Control & Pre-Manifest)	(Days)
1	0.80	0.76	3.45
2	0.84	0.81	3.78
3	0.88	0.86	4.12
4	0.92	0.81	4.61
5	0.88	0.76	5.82
6	0.88	0.71	6.17
7	0.84	0.71	7.56
8	0.80	0.67	8.43
9	0.80	0.67	9.62
10	0.84	0.62	10.38

**Table 2:** Forecasting accuracy results.The second column shows the results using control, pre-manifest and manifest cases while the third column includes only control and pre-manifest cases.

Field	Control & Manifest	Control
	& Pre-Manifest (%)	& Pre-Manifest (%)
Correct classification	0.92	0.86
Sensitivity	0.95	0.88
Specificity	0.80	0.80

## Data Availability

Access to the data and code is facilitated through a Github repository. https://github.com/Redbluelabel/HD.git. Last accessed on 1 March 2022.

## Supplementary Materials

The following supporting information can be downloaded at: https://www.mdpi.com/article/10.3390/jcm11082110/s1, Table S1: CpGs.
